# GJA1 promotes hepatocellular carcinoma progression by mediating TGF-β-induced activation and the epithelial–mesenchymal transition of hepatic stellate cells

**DOI:** 10.1515/med-2021-0344

**Published:** 2021-09-30

**Authors:** Gengming Niu, Xiaotian Zhang, Runqi Hong, Ximin Yang, Jiawei Gu, Tao Song, Zhiqing Hu, Liang Chen, Xin Wang, Jie Xia, Zhongwei Ke, Jun Ren, Liang Hong

**Affiliations:** Department of General Surgery, Shanghai Fifth People’s Hospital, Fudan University, Minhang District, Shanghai, 200240, People’s Republic of China; Department of Radiology, Dongying New District Hospital, Dongying, Shandong Province, 257000, People’s Republic of China; Department of General Surgery, Shanghai Fifth People’s Hospital, Fudan University, 801 Heqing Road, Minhang District, Shanghai, 200240, People’s Republic of China

**Keywords:** hepatocellular carcinoma, metastasis, hepatic stellate cell, TGF-β, epithelial–mesenchymal transition

## Abstract

**Introduction:**

Gap junction protein, alpha 1 (GJA1), which is correlated with recurrences and unfavorable prognoses in hepatocellular carcinomas (HCCs), is one of the specific proteins expressed by activated hepatic stellate cells (HSCs).

**Methods:**

Expression of GJA1 was compared between HCCs and nontumor tissues (NTs), between hepatic cirrhosis and NTs, and between primary and metastatic HCCs using transcriptomic datasets from the Gene Expression Omnibus and the Integrative Molecular Database of Hepatocellular Carcinoma. The *in vitro* activities of GJA1 were investigated in cultured HSCs and HCC cells. The underlying mechanism was characterized using Gene Set Enrichment Analysis and validated by western blotting.

**Results:**

The expression of GJA1 was significantly increased in HCCs and hepatic cirrhosis compared to that in NTs. GJA1 was also overexpressed in pulmonary metastases from HCCs when compared with HCCs without metastasis. Overexpression of GJA1 promoted while knockdown of GJA1 inhibited proliferation and transforming growth factor (TGF)-β-mediated activation and migration of cultured HSCs. Overexpression of GJA1 by lentivirus infection promoted proliferation and migration, while conditioned medium from HSCs overexpressing GJA1 promoted migration but inhibited proliferation of Hep3B and PLC-PRF-5 cells. Lentivirus infection with shGJA1 or conditioned medium from shGJA1-infected HSCs inhibited the proliferation and migration of HCCLM3 cells that had a high propensity toward lung metastasis. Mechanistically, GJA1 induced the epithelial–mesenchymal transition (EMT) in HSCs and HCCLM3 cells.

**Conclusion:**

GJA1 promoted HCC progression by inducing HSC activation and the EMT in HSCs. GJA1 is potentially regulated by TGF-β and thus may be a therapeutic target to inhibit HCC progression.

## 1 Introduction

Hepatocellular carcinoma (HCC) is one of the most predominant and fatal malignancies worldwide [1]. Although immunization with vaccines against the hepatitis B virus could dramatically decrease the incidence of HCC in high-risk populations [2], this malignancy remains a huge health threat due to the high probability of metastasis [3]. Therefore, it is crucial to characterize the underlying mechanisms of HCC metastasis to develop effective therapeutic measures and improve patient survival.

Activation of hepatic stellate cells (HSCs) initiates HCC recurrence and metastasis [4,5,6,7]. Stimulated by various profibrotic factors such as transforming growth factor (TGF)-β, HSCs are activated and transdifferentiated into myofibroblasts that secrete extracellular matrix proteins into the stroma to promote the development of hepatic fibrosis and cirrhosis, which are predisposed to and often interact with HCCs [7,8,9,10]. Activated HSCs also express a number of specific genes that facilitate HCC recurrence [4,5]. By inhibiting HSC activation and minimizing the adverse effects of profibrotic factors, fibrotic and cirrhotic liver diseases may be effectively treated [9,10,11], and HCC progression may be inhibited.

Gap junction protein, alpha 1 (GJA1), also known as connexin 43 (Cx43), belongs to the connexin family. It mediates the intercellular transmission of small molecules by means of gap junctional intercellular communication (GJIC) [12]. GJA1 mediates GJIC between neighboring HSCs and is upregulated in activated HSCs in response to inflammatory and proliferative stimuli [13]. GJA1 has been associated with hepatic fibrosis in rats and HCC progression in patients and cultured cells [5,13,14,15]. However, there are conflicting reports regarding whether GJA1 suppresses fibrosis and HCC progression [16,17,18]. To eliminate this controversy, it is important to investigate whether GJA1 is actively involved in HSC activation and whether this involvement is functionally implicated in HCC progression.

In the current study, we used public datasets to compare GJA1 expression between HCCs and adjacent nontumor tissues (NTs), between hepatic cirrhosis and NTs, as well as between metastatic HCCs and HCCs without metastasis. We then examined the *in vitro* effects of GJA1 on HSC activation with or without TGF-β stimulation. In addition, we evaluated the direct and indirect effects of GJA1 on the proliferation and migration of cultured HCC cells. Finally, we characterized the underlying mechanism of action of GJA1.

## 2 Materials and methods

### 2.1 Access to public datasets and the Gene Set Enrichment Analysis (GSEA)

Thirteen datasets including GSE6764 [19], GSE10143 [20], GSE14323 [21], GSE14520 [22,23], GSE22058 [24,25], GSE25097 [26,27,28], GSE36376 [29], GSE46444 [30], GSE54236 [31], GSE63898 [32], GSE76427 [33], the Liver Hepatocellular Carcinoma Project of The Cancer Genome Atlas (TCGA-LIHC), 1The results published or shown here are in whole or part based upon data generated by the TCGA Research Network (http://cancergenome.nih.gov/). and the Liver Cancer-RIKEN, JP Project from the International Cancer Genome Consortium [34] were retrieved from the Gene Expression Omnibus [35] and the Integrative Molecular Database of Hepatocellular Carcinoma [36], and analyzed for GJA1 mRNA expression in cirrhosis, HCC, and hepatic NTs. These datasets included 1,438 NTs, 2,061 HCCs, and 94 cirrhotic liver tissues. In addition, two datasets of primary and metastatic HCCs were downloaded from the Gene Expression Omnibus (GSE40367 [37] and GSE364 [38]). To explore potential mechanisms of GJA1 in HCC progression, a GSEA was employed using the TCGA-LIHC dataset.

### 2.2 Cell culture and lentivirus infection

The HSC-LX2 human hepatic stellate cell line was obtained from Shanghai Ke Lei Biological Technology (Shanghai, China). HCCLM3 cells were kind gifts from Dr. Lijie Ma from the Liver Cancer Institute, Zhongshan Hospital, Fudan University, Shanghai, China. Hep3B, PLC-PRF-5, and SK-Hep1 cells were provided by Prof. Yongzhong Liu from the Shanghai Cancer Institute, Shanghai, China. All cell lines used in this study have been validated by short tandem repeat profiling methods. Cells were cultured in Dulbecco’s modified essential medium (DMEM; BBI Life Sciences, Shanghai, China) supplemented with 10% fetal bovine serum (FBS), 100 µg/mL penicillin, and 100 mg/mL streptomycin at 37°C with 5% CO_2_ in a humidified incubator (Thermo Fisher Scientific, Waltham, MA, USA). GFP-expressing lentivirus particles were prepared by Genechem (Shanghai, China). The vectors used were GV358 (for GJA1 and the vector control) and GV248 (for shGJA1 and the scrambled control). The target sequence for shGJA1 was CCAAACTGATGGTGTCAAT. The titration was 2E + 9 (TU/mL) for all types of lentiviruses.

### 2.3 Collection of conditioned medium (CM)

The CM from LX2 cells was collected as previously described [39]. Briefly, stably transfected cells (1 × 10^6^) were seeded into 100 mm dishes containing 10 mL of DMEM with 10% FBS for 24 h and washed twice with serum-free DMEM. The cells were cultured in serum-free DMEM for another 24 h, and the supernatants were collected, centrifuged, filtered, and stored at −20°C until use.

### 2.4 Treatment of HSCs with recombinant human TGF-β1

HSCs were seeded in 100 mm dishes and 96-, 12-, and 6-well plates at the indicated concentrations with 10% FBS in the medium. After 24 h, the cells were serum-starved for 24 h and treated with recombinant hTGF-β1 (Univ-Bio, Shanghai, China) at a final concentration of 5 ng/mL, or with an equal volume of phosphate-buffered saline (PBS) as control and incubated for 24, 48, 72, or 96 h.

### 2.5 RNA extraction and quantitative polymerase chain reaction (qPCR)

Total RNA was isolated from cell cultures using RNAiso Plus (Takara Bio, Kusatsu, Japan) according to the manufacturer’s instructions, and reverse-transcribed and subjected to real-time reverse transcription-PCR using the 2^−ΔΔCT^ method. The sequences for RT-PCR primers were the following: GJA1 forward primer, 5′-CAATCTCTCATGTGCGCTTCT-3′; and GJA1 reverse primer, 5′-GGCAACCTTGAGTTCTTCCTCT-3′. Glyceraldehyde 3-phosphate dehydrogenase (GAPDH) served as an internal control. Experiments were repeated three times, in duplicate.

### 2.6 Western blotting

Total protein was extracted from cell cultures at the indicated time points using radioimmunoprecipitation assay lysis buffer (Beyotime Biotechnology, Shanghai, China) containing phenylmethylsulfonyl fluoride (Beyotime Biotechnology) and proteinase inhibitor cocktail solution (Roche, Basel, Switzerland), and quantitated using the bicinchoninic acid protein assay (Beyotime Biotechnology) as recommended by the manufacturers. Western blotting was performed as previously described [40] using rabbit anti-GJA1 polyclonal antibody (1:1,000 dilution; Univ-Bio) or a mouse anti-α-smooth muscle actin (SMA) monoclonal antibody (1:1,000 dilution; Univ-Bio). Other antibodies used in this study are listed in Supplementary Table A1. GAPDH (1:2,000 dilution, rabbit anti-human; Beyotime Biotechnology) was detected as a loading control. The grayscale values of protein bands were analyzed using ImageJ software (National Institutes of Health, Bethesda, MD, USA).

### 2.7 Proliferation assay

Stably transfected LX2, Hep3B, PLC-PRF-5, or HCCLM3 cells (2 × 10^3^ cells/well) were seeded in 96-well plates with 10% FBS in the medium and cultivated for 24 h and then serum-starved for another 24 h. TGF-β1 or an equal volume of PBS was added and the cells were cultivated for 24, 48, 72, or 96 h. Then, 10 μL of cholecystokinin octapeptide-8 reagent [10% (v/v) in serum-free DMEM; Beyotime Biotechnology] was added to each well and incubated at 37°C for 1 h. The absorbance at 450 nm was measured using a microplate reader (BioTek Synergy 2; BioTek, Winooski, VT, USA).

### 2.8 Migration assay

Stably transfected LX2, Hep3B, PLC-PRF-5, or HCCLM3 cells (4 × 10^5^ cells/mL) were seeded in serum-free DMEM with or without TGF-β1 in the top chamber of a Transwell insert. The medium containing 20% FBS in the lower chamber served as a chemoattractant. After incubation for 24 h at 37°C, the cells on the top side of the membrane were removed with a cotton swab, and those on the bottom side were fixed with methanol for 20 min and stained with crystal violet (0.1% in PBS) for 15 min. Six randomly selected fields per well were photographed, and the numbers of migrated cells were counted.

### 2.9 Scratch wound healing assay

A monolayer scratch wound assay was conducted as previously described [41]. Briefly, stably transfected LX2 cells (4 × 10^5^ cells/well) were seeded in 12-well plates with 10% FBS in the medium and grown to nearly 100% confluence. After 24 h of serum starvation, a scratch wound was generated with a 200 μL pipette tip. Wound closure was photographed at 0, 24, and 48 h.

### 2.10 Statistical analysis

Analyses were performed using Microsoft Excel 2010 (Microsoft, Redmond, WA, USA) or GraphPad Prism7 (GraphPad, San Diego, CA, USA). Student’s *t-*test or one-way analysis of variance was performed for continuous variables. Statistical significance was defined as a value of *p <* 0.05. All statistical tests were two-sided.

## 3 Results

### 3.1 GJA1 is upregulated in HCCs and hepatic cirrhosis

**Figure 1:**
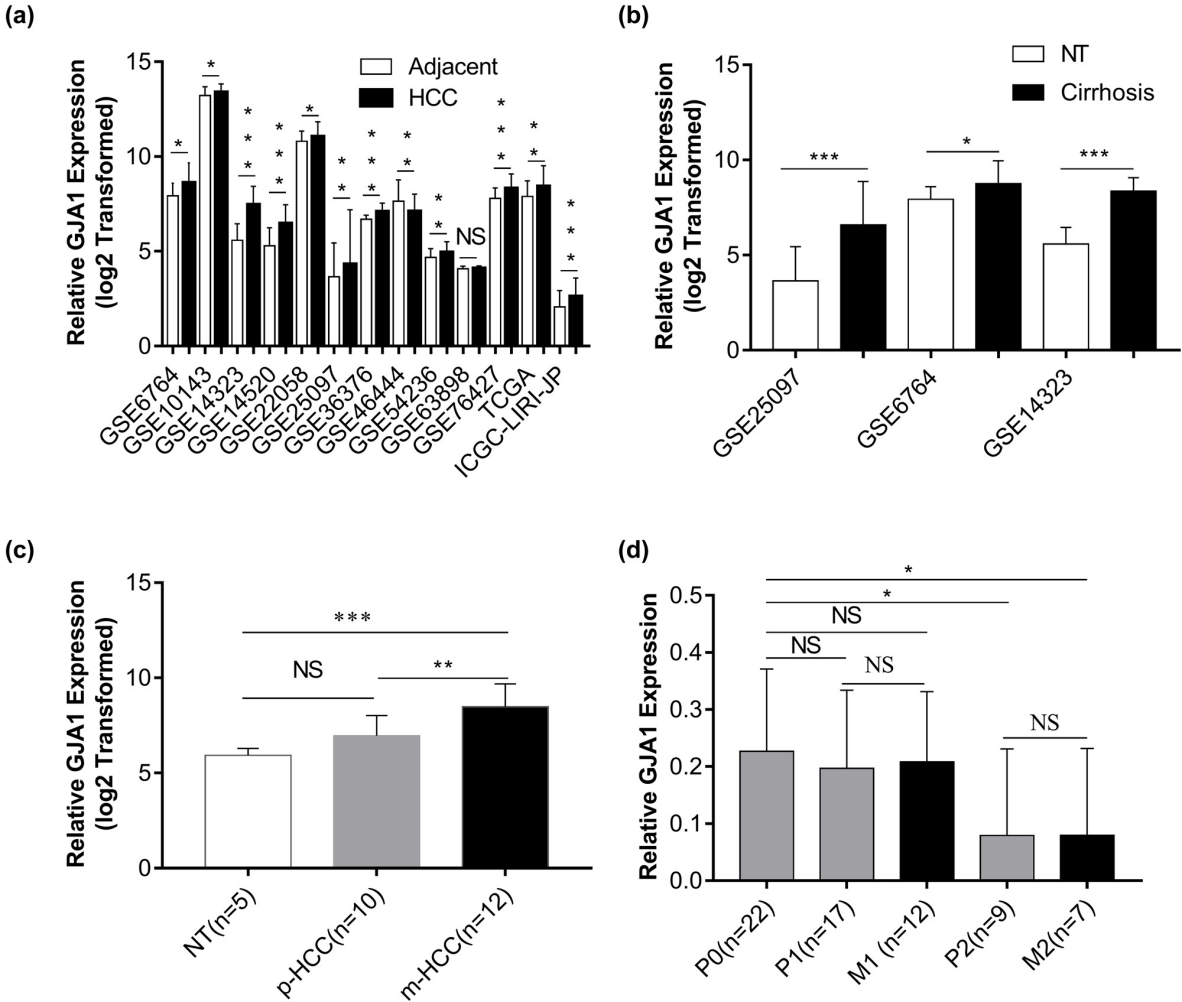
GJA1 is upregulated in HCCs and hepatic cirrhosis. The expression of GJA1 was compared between HCCs and adjacent NTs (a), between hepatic cirrhosis and NTs (b), between pulmonary metastases from HCCs (m-HCC) and primary HCCs without metastasis (p-HCC) (c), and between HCCs with intrahepatic metastasis (M1) or with portal vein tumor thrombus metastases (M2) and their corresponding primary HCCs (P1 or P2) or primary HCCs without metastasis (P0) (d). GJA1, gap junction protein, alpha 1; HCC, hepatocellular carcinoma; NS, not significant; NT, nontumor tissue; *p < 0.05; **p < 0.01; ***p < 0.001 vs the control group.

Because the functional relevance of GJA1 in HCCs and hepatic cirrhosis remains unknown, we analyzed GJA1 mRNA expression with 13 public datasets. Figure 1a shows that in HCCs compared with adjacent NTs, GJA1 was significantly upregulated in 11 datasets, significantly downregulated in one dataset, and was similar in one dataset. In three of the datasets with cirrhosis samples, GJA1 expression was significantly increased in hepatic cirrhosis compared with that in NTs (Figure 1b). Surprisingly, by analyzing one dataset consisting of 5 NTs, 10 primary HCCs without metastasis (p-HCC), and 12 pulmonary metastases from HCCs (m-HCC), we found that GJA1 expression was significantly increased in pulmonary metastases from HCCs compared to that in NTs or primary HCCs without metastasis (Figure 1c). By contrast, in another dataset that contained 22 primary HCCs without metastasis (P0), 17 primary lesions (P1) and 12 metastases (M1) from HCCs with intrahepatic spread metastasis, and 9 primary lesions (P2) and 7 metastases (M2) from HCCs with portal vein tumor thrombus metastasis, the expression of GJA1 was significantly decreased in P2 and M2 samples while decreased with no significance in P1 and M1 samples, compared to that in P0 samples (Figure 1d). Besides, GJA1 expression was quite similar between primary tumor lesions and their matched distant metastases (Figure 1d). Taken together, these results indicated that GJA1 transcription was enhanced in HCCs, hepatic cirrhosis, and pulmonary metastases from HCCs, but diminished in portal vein tumor thrombus metastasis from HCCs, compared to their relative controls.

### 3.2 TGF-β-mediated HSC activation and migration are dependent on GJA1

**Figure 2:**
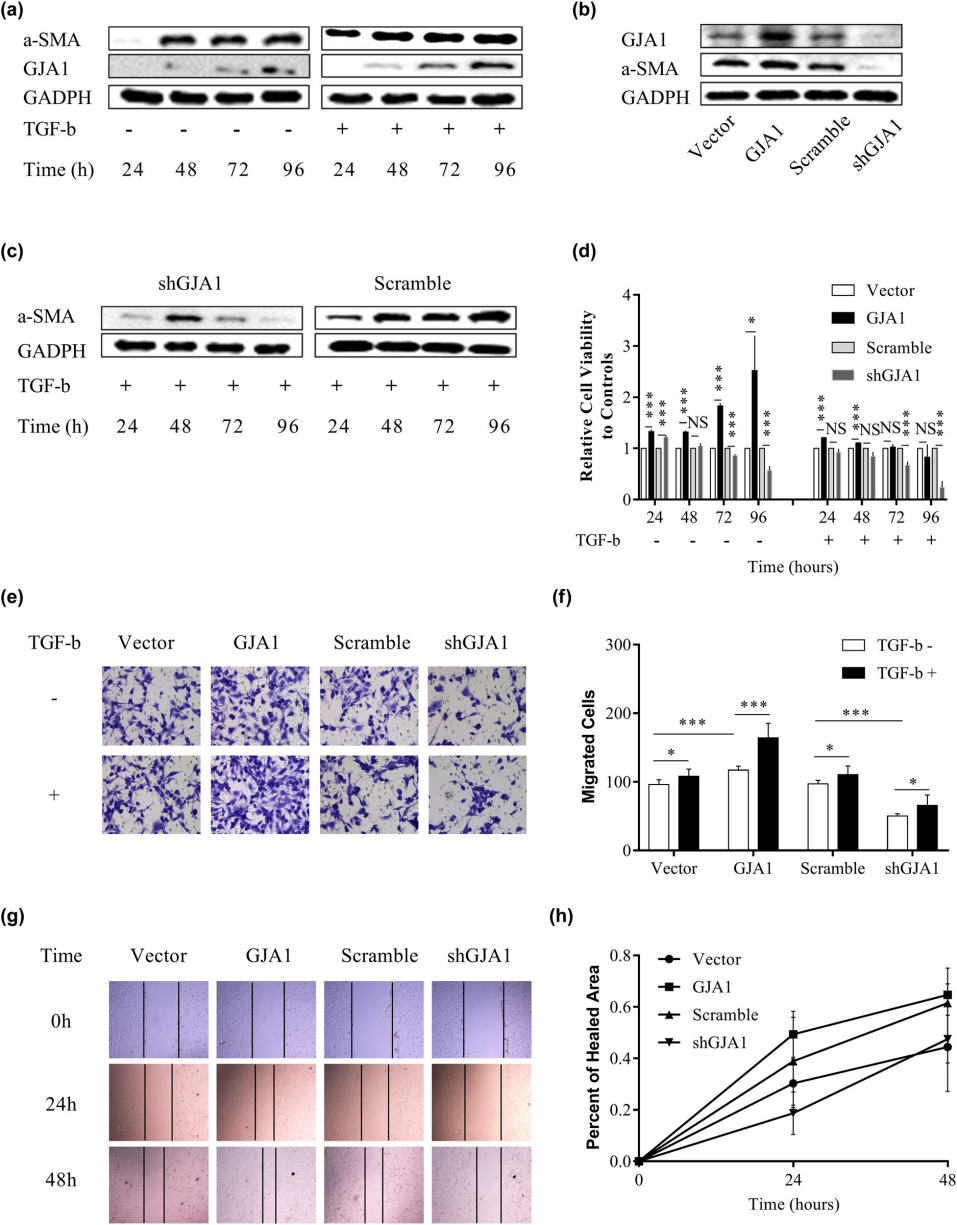
TGF-β-mediated HSC activation and migration are dependent on GJA1. LX2 cells were stimulated with or without TGF-β for 24, 48, 72, and 96 h, and cell lysates were collected and analyzed using western blotting with the indicated antibodies (a). Cell lysates were collected from LX2 stable infectants expressing GJA1, vector control, shGJA1, or scrambled control, followed by western blotting using the indicated antibodies (b). LX2 stable infectants expressing shGJA1 or scrambled control were stimulated with TGF-β for 24, 48, 72, and 96 h, and cell lysates were collected and analyzed by western blotting with the indicated antibodies (c). LX2 stable infectants expressing GJA1, vector control, shGJA1, or scrambled control were seeded into 96-well plates and stimulated with or without TGF-β for 24, 48, 72, and 96 h, followed by cholecystokinin octapeptide-8 assays (d). LX2 stable infectants expressing GJA1, vector control, shGJA1, or scrambled control were stimulated with or without TGF-β for 24 h and analyzed using the Transwell migration assay (e and f). LX2 stable infectants expressing GJA1, vector control, shGJA1, or scrambled control were seeded into 12-well plates and analyzed using the scratch wound-healing assay. Wound closure was photographed at 0, 24, and 48 h and quantitated (g and h). α-SMA, alpha smooth muscle actin; TGF-β, transforming growth factor-β; HSC, hepatic stellate cell; GJA1, gap junction protein, alpha 1.

HSC activation is the key contributing factor of hepatic cirrhosis and HCC progression, characterized by enhanced HSC proliferation, migration, contraction, and expression of specific markers such as alpha smooth muscle actin (α-SMA) [7,42]. TGF-β, the most potent fibrogenic cytokine, promotes HSC activation and HCC progression [42,43]. We therefore investigated whether TGF-β-mediated HSC activation was dependent on GJA1. Figure 2a shows that HSCs became activated 48 h after resuscitation, which was accelerated by TGF-β1 stimulation and coincided with elevated GJA1 expression. Ectopic expression of GJA1 upregulated while knockdown of GJA1 downregulated α-SMA expression in HSCs (Figure 2b). In addition, knockdown of GJA1 significantly abrogated TGF-β1-induced α-SMA overexpression at 72 and 96 h (Figure 2c). We further evaluated whether GJA1 influenced HSC proliferation and migration. Figure 2d shows that overexpression of GJA1 significantly promoted HSC proliferation, which was most apparent at 72 and 96 h, while knockdown of GJA1 remarkably suppressed HSC proliferation at 72 and 96 h. Concurrent TGF-β1 treatment with GJA1 overexpression had no effects on HSC proliferation at 72 and 96 h, whereas concurrent TGF-β1 treatment with GJA1 knockdown further inhibited HSC proliferation at these time points. By contrast, overexpression of GJA1 accelerated and knockdown of GJA1 inhibited HSC migration, while concurrent TGF-β1 treatment increased the effects of GJA1 overexpression and neutralized the effects of GJA1 knockdown on HSC migration, as suggested by the Transwell migration assay (Figure 2e and f). Similarly, the wound healing assay also confirmed that GJA1 promoted the migration of HSCs (Figure 2g and h). Taken together, these results indicated that GJA1 was a potential downstream target of TGF-β that was necessary for TGF-β-induced HSC activation and migration but not proliferation.

### 3.3 GJA1 differentially affects the proliferation and migration of cultured HCC cells

**Figure 3:**
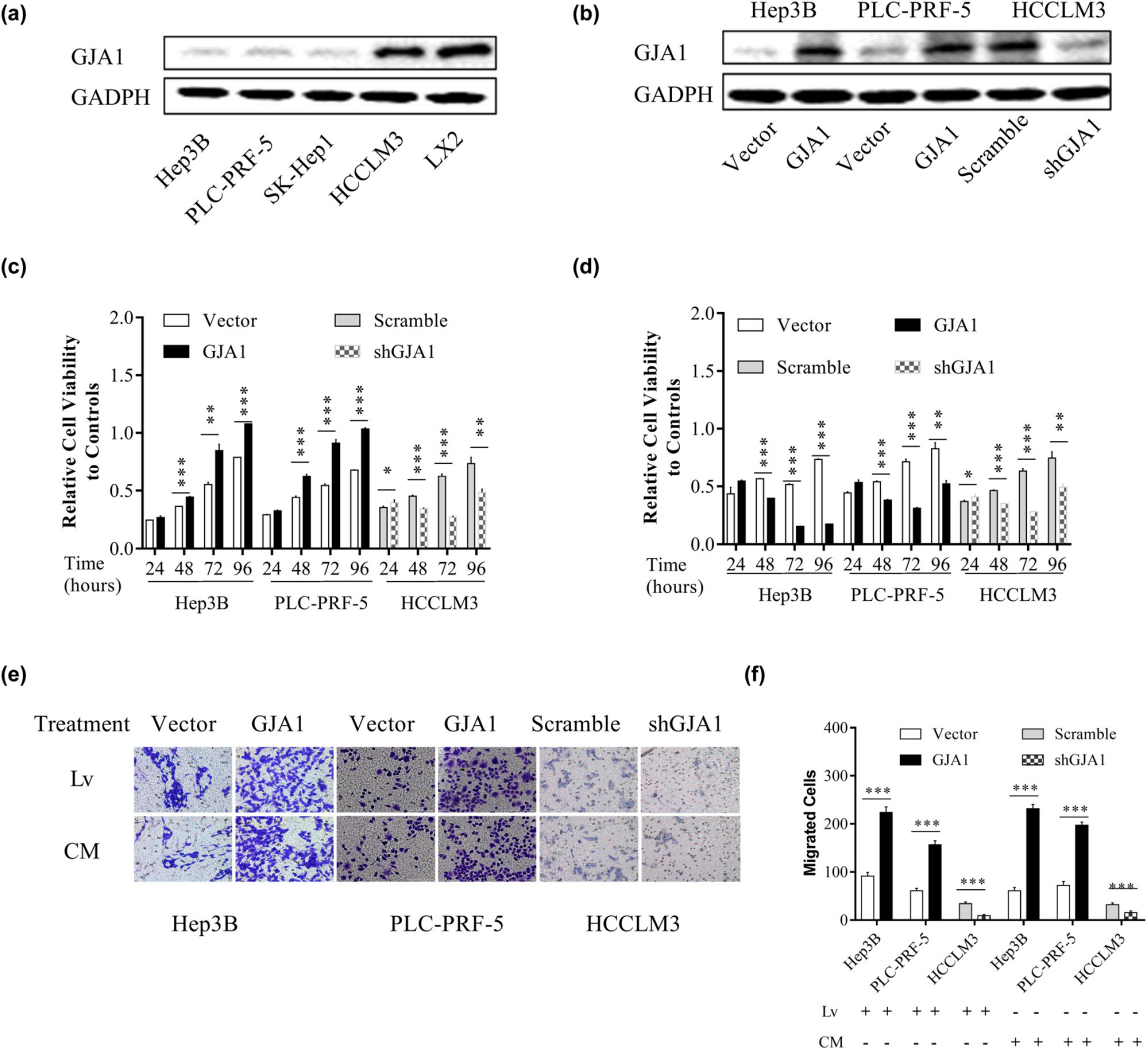
GJA1 differentially affects the proliferation and migration of cultured HCC cells. The intrinsic expression of GJA1 in cultured HCC cells and HSCs was analyzed by western blotting (a). HEP3B and PLC-PRF-5 cells with low to moderate malignant potencies were transduced with lentiviruses carrying GJA1 or vector control, while HCCLM3 cells with a high tendency for pulmonary metastasis were transduced with shGJA1 or scrambled control. Cell lysates were analyzed by western blotting (b). HCC cells transduced with lentiviruses expressing GJA1, vector control, shGJA1, or scramble control were seeded into 96-well plates and analyzed using the cholecystokinin octapeptide-8 assay (c). HCC cells stimulated with conditioned medium from HSCs transduced with the indicated lentiviruses were seeded into 96-well plates and analyzed using the cholecystokinin octapeptide-8 assay (d). HCC cells transduced with the indicated lentiviruses or stimulated with CM from HSCs transduced with indicated lentiviruses were analyzed using the Transwell migration assay (e and f). CM, conditioned medium; GJA1, gap junction protein, alpha 1; HCC, hepatocellular carcinoma; HSC, hepatic stellate cell; Lv, lentiviruses.

To further characterize the role of GJA1 in HCC progression, we evaluated the effects of GJA1 on the proliferation and migration of cultured HCC cells. We first assessed the abundance of GJA1 in four HCC cell lines with different malignant potencies and LX2. Figure 3a shows that GJA1 was significantly downregulated in Hep3B, PLC-PRF-5, and SK-Hep1 cells that had low to moderate malignant potencies, compared to that in LX2 cells and HCCLM3 cells that were derived from MHCC97-H cells and had a high tendency for pulmonary metastasis. We then overexpressed GJA1 in Hep3B and PLC-PRF-5 cells and knocked down GJA1 in HCCLM3 cells (Figure 3b). GJA1 overexpression significantly increased the proliferation of HEP3B and PLC-PRF-5 cells, while GJA1 knockdown significantly decreased the proliferation of HCCLM3 cells, from 48 to 96 h (Figure 3c). Culturing with the corresponding CM from HSCs yielded similar results in HCCLM3 cells but opposite results in HEP3B and PLC-PRF-5 cells, compared to that in lentivirus-transduced cells (Figure 3d). GJA1 overexpression significantly accelerated the migration of HEP3B and PLC-PRF-5 cells, and GJA1 knockdown significantly impeded the migration of HCCLM3 cells, while cultivation with the corresponding CM from HSCs yielded similar results in these cells compared with that in lentivirus-transduced cells (Figure 3e and f). Collectively, these results suggested that the activity of GJA1 was cell type-dependent.

### 3.4 GJA1 induces the epithelial–mesenchymal transition (EMT)

**Figure 4:**
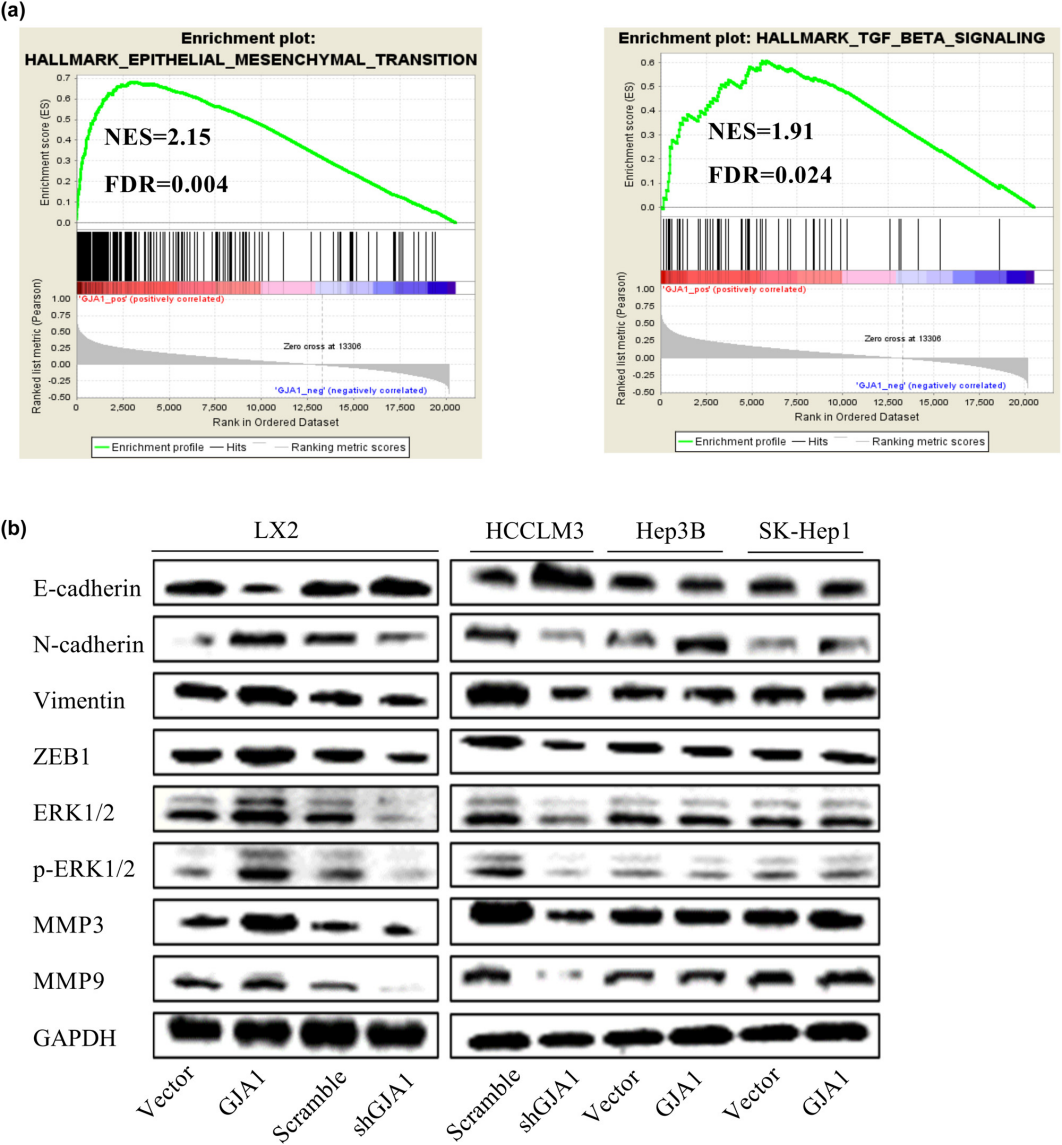
GJA1 induces the EMT. A GSEA indicated that GJA1 expression was positively correlated with the hallmark gene sets EPITHELIAL_MESENCHYMAL_TRANSITION (a, left) and TGF_BETA_SIGNALING (a, right). Cell lysates were collected from LX2 stable infectants expressing GJA1, vector control, shGJA1, or scrambled control cells, followed by western blot analyses with the indicated antibodies (b, left panel). HEP3B and PLC-PRF-5 cells were transduced with lentiviruses carrying GJA1 or vector control, while HCCLM3 cells were transduced with shGJA1 or the scrambled control. Cell lysates were collected and were analyzed using western blotting with the indicated antibodies (b, right panel). EMT, epithelial–mesenchymal transition; ERK, extracellular regulated protein kinases; FDR, false discovery rate; GJA1, gap junction protein, alpha 1; GSEA, Gene Set Enrichment Analysis; MMP, matrix metalloproteinase; NES, normalized enrichment score; ZEB1, zinc finger E-box-binding homeobox 1.

To characterize the potential underlying mechanism of GJA1 activity, we first performed a GSEA using the TCGA-LIHC dataset. We found that high GJA1 expression was positively correlated with the hallmark gene sets EPITHELIAL_MESENCHYMAL_TRANSITION and TGF_BETA_SIGNALING (Figure 4a) as well as several other gene sets that were closely associated with tumor progression (data not shown). To verify these observations, we tested the effects of GJA1 on several EMT markers and EMT-related transcription factors in LX2, HCCLM3, PLC-PRF-5, and HEP3B cells transduced with the indicated lentiviruses. As the ERK1/2 pathway and MMPs are critically involved in TGF-β-mediated EMT [44,45], we also tested the effects of GJA1 on key molecules in these pathways. Western blot analysis showed that GJA1 overexpression in HSCs decreased the epithelial marker E-cadherin, increased the mesenchymal markers N-cadherin, vimentin, and Zeb1, activated the ERK1/2-MAPK signaling pathway, and increased MMP-3 and MMP-9 compared to control cells. GJA1 knockdown in HSCs and HCCLM3 cells increased E-cadherin, decreased N-cadherin, vimentin, and Zeb1, inhibited the ERK1/2-MAPK signaling pathway, and decreased MMP-3 and MMP-9 compared to control cells. By contrast, GJA1 overexpression in PLC-PRF-5 and HEP3B cells had no consistent effects on these markers (Figure 4b). Overall, these results suggested that GJA1 promoted HCC progression by inducing the EMT in both HSCs and highly aggressive HCC cells.

## 4 Discussion

GJA1 is one of the specific proteins expressed by activated HSCs and is positively associated with HCC recurrence and unfavorable prognoses [5]. GJA1 also mediates hepatic cirrhosis and metastasis of several malignancies [15,46]. Consistent with these observations, the present study showed that GJA1 was almost always overexpressed in HCCs and hepatic cirrhosis, compared to corresponding NTs. In addition, GJA1 expression was significantly increased in lung metastases from HCCs and HCCLM3 cells that had a high propensity for lung metastasis, compared to HCCs without metastasis and HCC cells with less malignant potentials, which is highly consistent with observations made by Ogawa et al. using a rat model of HCC [47]. Furthermore, the overexpression of GJA1 promoted, while knockdown of GJA1 inhibited, the proliferation and migration of cultured HCC cells. Unexpectedly, we found that GJA1 expression was decreased significantly in portal vein tumor thrombus metastases, while it was decreased with no significance in intrahepatic metastases compared to that in corresponding primary HCCs. As primary tumor lesions and their matched distant metastases share large similarities at the genomic and transcriptomic levels, the metastatic propensity of the primary tumor has been suggested as “inherent” [37]; thus, the enhanced GJA1 expression in primary tumors could indicate a high propensity of pulmonary metastasis. To our surprise, CM from HSCs overexpressing GJA1 differentially influenced the proliferation and migration of HCC cells with relatively lower malignant potency. Although CM from activated HSCs generally promotes HCC progression [48,49,50], conflicting results were occasionally reported in the literature [51,52]. Besides, Coulouar et al. identified the bidirectional crosstalk between HSCs and HCC cells, which induced HCC cell migration rather than proliferation, and generation of the proangiogenic microenvironment in HSC-LX2 cells [8]. Thus the inconsistency observed in Hep3B and PLC-PRF-5 cells may be ascribed to different pathways mediating proliferation and migration of HCC cells that are targeted by proteins secreted by GJA1-overexpressing HSCs. Together, these results suggested that the roles of GJA1 were tumor-type and cell type-dependent, and could partly explain the discrepancy in previous reports that GJA1 had contrasting effects on tumor progression.

Among various profibrotic factors, TGF-β is generally considered the most potent. Binding of TGF-β to the type I receptor induces phosphorylation of the receptor, subsequently resulting in phosphorylation and activation of the factor, and small molecules targeted against decapentaplegic-3, followed by enhanced transcription of type I and type III collagen [42]. TGF-β also promotes HSC activation by activating the MAPK signaling pathway, including extracellular signal-regulated kinase, p38, and c-jun N-terminal kinase [42]. In the present study, we found that GJA1 was upregulated by TGF-β1 in cultured and activated human HSCs, and its presence was necessary for TGF-β1-induced HSC activation and migration, but not for TGF-β1-mediated proliferation inhibition. However, conflicting reports from previous studies have suggested that the regulation of GJA1 by TGF-β is highly diversified. For example, TGF-β upregulated GJA1 expression in human granulosa cells and ovarian cancer cells [53,54] but downregulated GJA1 expression in rat HSCs [55]. This may partly be explained by the observation that GJA1-mediated GJIC was organ-specific and only existed between neighboring HSCs but not between HSCs and other cell types in the liver [13], and that the plasma membrane localization of GJA1 was functional, promoting disease metastases, while the cytoplasmic localization was not functional, accompanied by reduced cell proliferation, adhesion, and invasion [46]. Besides, the identification of N-terminally truncated GJA1 isoforms may further complicate this case, as the most predominant one GJA1 20k regulates GJA1 gap junction formation and inhibition of this isoform leads to redistribution and malfunction of GJA1 [56]. In addition, being one of the specific genes expressed by activated HSCs [5], GJA1 promoted HSC activation in return, which suggests positive feedback between HSC activation and GJA1 overexpression that leads to augmented GJIC between HSCs. These observations underline the complicacy and importance of GJA1 in mediating tumor progression and need to be addressed in future studies.

In the current study, the GSEA results showed that high GJA1 expression was positively correlated with the hallmark gene sets EPITHELIAL_MESENCHYMAL_ TRANSITION and TGF_BETA_SIGNALING as well as several other tumor-related gene sets, providing further evidence that GJA1 is closely associated with tumor progression in HCCs. The EMT is one of the key processes mediating cirrhosis and tumor metastasis and has been classified into three different biological subtypes based on its biological context. Type 1 EMTs generate primary mesenchymal cells and are related to implantation, embryo formation, and organ development. Type 2 EMTs are involved in wound healing, tissue regeneration, and organ fibrosis. Type 3 EMTs occur in neoplastic cells and induce cancer invasion and metastasis [45]. TGF-β induces the EMT in both fibrosis and cancer and has been suggested as a target to reverse the EMT [44]. Our results showed that GJA1 induced the EMT in LX2 and HCCLM3 cells but not in PLC-PRF-5 or HEP3B cells, which may contribute to its functional complexity in HCC progression. Stimulated by TGF-β, normal mouse mammary gland epithelial cells undergo EMT with increased expression of full-length GJA1 but reduced expression of GJA1 20k and gap junction formation [56]. However, as enhanced gap junction formation may be associated with increased HSC activation, these observations once again demonstrate the tumor-type and cell type-dependent roles of GJA1.

The current study presents some findings that are clinically and scientifically meaningful, which may help better understand the critical involvement of GJA1 in hepatic fibrosis and HCC metastasis; however, there are some inherent limitations, including but not limited to the following. First, the pooled analyses of different datasets would bring great heterogeneity because of the largely variable background, which should be taken into consideration before any conclusions can be drawn. Second, we did not confirm the *in vivo* tumor-promoting activity of GJA1 using metastatic xenograft models since the cell lines we used failed to derive lung metastasis, maybe due to the short observation period. Third, although we uncovered the potential regulation of GJA1 on EMT of HSCs and HCC cells, we did not further elaborate on the underlying mechanism in the current study. Last, the transcriptional changes of GJA1 observed in different metastases from HCCs were only based on limited sample numbers, which should be validated in larger patient cohorts. These limitations should be addressed in future studies.

## 5 Conclusion

In summary, we have identified GJA1 as a potential downstream target of TGF-β that may promote HCC progression by mediating TGF-β-induced activation and the EMT of HSCs.

## Abbreviations

CM: conditioned medium
Cx43: connexin 43
EMT: epithelial–mesenchymal transition
GJA1: gap junction protein, alpha 1
GJIC: gap junctional intercellular communication
GSEA: Gene Set Enrichment Analysis
HCC: hepatocellular carcinoma
HSC: hepatic stellate cell
LIHC: the Liver Hepatocellular Carcinoma Project
NT: normal tissue
PBS: phosphate-buffered saline
q-PCR: quantitative polymerase chain reaction
SMA: α-smooth muscle actin
TCGA: The Cancer Genome Atlas
TGF: transforming growth factor

## Appendix

**Table A1:** Antibody information datasheet^*^

Protein ID	Species	Purification	Cat. no.	Producer
Vimentin	Rabbit	mAb	abs130228	Absin Biotechnology, Shanghai, China
MMP3	Rabbit	mAb	14351S	Cell Signaling Technology, Danvers, MA, USA
MMP9	Rabbit	mAb	13667	Cell Signaling Technology, Danvers, MA, USA
ERK1/2	Rabbit	mAb	4695	Cell Signaling Technology, Danvers, MA, USA
p-ERK1/2	Rabbit	mAb	4376	Cell Signaling Technology, Danvers, MA, USA
E-cadherin	Rabbit	mAb	3195	Cell Signaling Technology, Danvers, MA, USA
N-cadherin	Rabbit	mAb	13116	Cell Signaling Technology, Danvers, MA, USA
Zeb1	Rabbit	mAb	3396	Cell Signaling Technology, Danvers, MA, USA

^*^All antibodies were diluted at 1:1,000.

## References

[1] Sung H, Ferlay J, Siegel RL, Laversanne M, Soerjomataram I, Jemal A, et al. Global cancer statistics 2020: GLOBOCAN estimates of incidence and mortality worldwide for 36 cancers in 185 countries. CA Cancer J Clin. 2021;71(3):209–49. 10.3322/caac.21660. 33538338

[2] Chang MH, Chen CJ, Lai MS, Hsu HM, Wu TC, Kong MS, et al. Universal hepatitis B vaccination in Taiwan and the incidence of hepatocellular carcinoma in children. Taiwan Childhood Hepatoma Study Group. N Engl J Med. 1997;336(26):1855–9. 10.1056/NEJM199706263362602. 9197213

[3] Siegel RL, Miller KD, Fuchs HE, Jemal A. Cancer statistics, 2021. CA Cancer J Clin. 2021;71(1):7–33. 10.3322/caac.21654. 33433946

[4] Zhang DY, Goossens N, Guo J, Tsai MC, Chou HI, Altunkaynak C, et al. A hepatic stellate cell gene expression signature associated with outcomes in hepatitis C cirrhosis and hepatocellular carcinoma after curative resection. Gut. 2016;65(10):1754–64. 10.1136/gutjnl-2015-309655. PMC4848165 26045137

[5] Ji J, Eggert T, Budhu A, Forgues M, Takai A, Dang H, et al. Hepatic stellate cell and monocyte interaction contributes to poor prognosis in hepatocellular carcinoma. Hepatology. 2015;62(2):481–95. 10.1002/hep.27822. PMC4515211 25833323

[6] Dou C, Liu Z, Tu K, Zhang H, Chen C, Yaqoob U, et al. P300 acetyltransferase mediates stiffness-induced activation of hepatic stellate cells into tumor-promoting myofibroblasts. Gastroenterology. 2018;154(8):2209–21.e14. 10.1053/j.gastro.2018.02.015. PMC6039101 29454793

[7] Thompson AI, Conroy KP, Henderson NC. Hepatic stellate cells: central modulators of hepatic carcinogenesis. BMC Gastroenterol. 2015;15:63. 10.1186/s12876-015-0291-5. PMC4445994 26013123

[8] Coulouarn C, Corlu A, Glaise D, Guenon I, Thorgeirsson SS, Clement B. Hepatocyte-stellate cell cross-talk in the liver engenders a permissive inflammatory microenvironment that drives progression in hepatocellular carcinoma. Cancer Res. 2012;72(10):2533–42. 10.1158/0008-5472. PMC3498759 22419664

[9] Mikuriya Y, Tashiro H, Kuroda S, Nambu J, Kobayashi T, Amano H, et al. Fatty liver creates a pro-metastatic microenvironment for hepatocellular carcinoma through activation of hepatic stellate cells. Int J Cancer. 2015;136(4):E3–13. 10.1002/ijc.29096. 25053237

[10] Coulouarn C, Clement B. Stellate cells and the development of liver cancer: therapeutic potential of targeting the stroma. J Hepatol. 2014;60(6):1306–9. 10.1016/j.jhep.2014.02.003. 24530649

[11] Faggioli F, Palagano E, Di Tommaso L, Donadon M, Marrella V, Recordati C, et al. B lymphocytes limit senescence-driven fibrosis resolution and favor hepatocarcinogenesis in mouse liver injury. Hepatology. 2018;67(5):1970–85. 10.1002/hep.29636. 29105104

[12] Evans WH. Cell communication across gap junctions: a historical perspective and current developments. Biochem Soc Trans. 2015;43(3):450–9. 10.1042/BST20150056. 26009190

[13] Fischer R, Reinehr R, Lu TP, Schonicke A, Warskulat U, Dienes HP, et al. Intercellular communication via gap junctions in activated rat hepatic stellate cells. Gastroenterology. 2005;128(2):433–48. 10.1053/j.gastro.2004.11.065. 15685554

[14] Zhang D, Kaneda M, Nakahama K, Arii S, Morita I. Connexin 43 expression promotes malignancy of HuH7 hepatocellular carcinoma cells via the inhibition of cell-cell communication. Cancer Lett. 2007;252(2):208–15. 10.1016/j.canlet.2006.12.024. 17275998

[15] Balasubramaniyan V, Dhar DK, Warner AE, Vivien Li WY, Amiri AF, Bright B, et al. Importance of Connexin-43 based gap junction in cirrhosis and acute-on-chronic liver failure. J Hepatol. 2013;58(6):1194–200. 10.1016/j.jhep.2013.01.023. 23376361

[16] Cogliati B, Da Silva TC, Aloia TP, Chaible LM, Real-Lima MA, Sanches DS, et al. Morphological and molecular pathology of CCL4-induced hepatic fibrosis in connexin43-deficient mice. Microsc Res Tech. 2011;74(5):421–9. 10.1002/jemt.20926. 20830702

[17] Naus CC, Laird DW. Implications and challenges of connexin connections to cancer. Nat Rev Cancer. 2010;10(6):435–41. 10.1038/nrc2841. 20495577

[18] Wang ZS, Wu LQ, Yi X, Geng C, Li YJ, Yao RY. Connexin-43 can delay early recurrence and metastasis in patients with hepatitis B-related hepatocellular carcinoma and low serum alpha-fetoprotein after radical hepatectomy. BMC Cancer. 2013;13:306. 10.1186/1471-2407-13-306. PMC3704436 23800357

[19] Wurmbach E, Chen YB, Khitrov G, Zhang W, Roayaie S, Schwartz M, et al. Genome-wide molecular profiles of HCV-induced dysplasia and hepatocellular carcinoma. Hepatology. 2007;45(4):938–47. 10.1002/hep.21622. 17393520

[20] Hoshida Y, Villanueva A, Kobayashi M, Peix J, Chiang DY, Camargo A, et al. Gene expression in fixed tissues and outcome in hepatocellular carcinoma. N Engl J Med. 2008;359(19):1995–04. 10.1056/NEJMoa0804525. PMC2963075 18923165

[21] Mas VR, Maluf DG, Archer KJ, Yanek K, Kong X, Kulik L, et al. Genes involved in viral carcinogenesis and tumor initiation in hepatitis C virus-induced hepatocellular carcinoma. Mol Med. 2009;15(3–4):85–94. 10.2119/molmed.2008.00110. PMC2605622 19098997

[22] Roessler S, Jia HL, Budhu A, Forgues M, Ye QH, Lee JS, et al. A unique metastasis gene signature enables prediction of tumor relapse in early-stage hepatocellular carcinoma patients. Cancer Res. 2010;70(24):10202–12. 10.1158/0008-5472. PMC3064515 21159642

[23] Roessler S, Long EL, Budhu A, Chen Y, Zhao X, Ji J, et al. Integrative genomic identification of genes on 8p associated with hepatocellular carcinoma progression and patient survival. Gastroenterology. 2012;142(4):957–66.e12. 10.1053/j.gastro.2011.12.039. PMC3321110 22202459

[24] Burchard J, Zhang C, Liu AM, Poon RT, Lee NP, Wong KF, et al. microRNA-122 as a regulator of mitochondrial metabolic gene network in hepatocellular carcinoma. Mol Syst Biol. 2010;6:402. 10.1038/msb.2010.58. PMC2950084 20739924

[25] Liu AM, Yao TJ, Wang W, Wong KF, Lee NP, Fan ST, et al. Circulating miR-15b and miR-130b in serum as potential markers for detecting hepatocellular carcinoma: a retrospective cohort study. BMJ Open. 2012;2(2):e000825. 10.1136/bmjopen-2012-000825. PMC3308260 22403344

[26] Tung EK, Mak CK, Fatima S, Lo RC, Zhao H, Zhang C, et al. Clinicopathological and prognostic significance of serum and tissue Dickkopf-1 levels in human hepatocellular carcinoma. Liver Int. 2011;31(10):1494–504. 10.1111/j.1478-3231.2011.02597.x. 21955977

[27] Lamb JR, Zhang C, Xie T, Wang K, Zhang B, Hao K, et al. Predictive genes in adjacent normal tissue are preferentially altered by sCNV during tumorigenesis in liver cancer and may rate limiting. PloS One. 2011;6(7):e20090. 10.1371/journal.pone.0020090. PMC3130029 21750698

[28] Sung WK, Zheng H, Li S, Chen R, Liu X, Li Y, et al. Genome-wide survey of recurrent HBV integration in hepatocellular carcinoma. Nat Genet. 2012;44(7):765–9. 10.1038/ng.2295. 22634754

[29] Lim HY, Sohn I, Deng S, Lee J, Jung SH, Mao M, et al. Prediction of disease-free survival in hepatocellular carcinoma by gene expression profiling. Ann Surg Oncol. 2013;20(12):3747–53. 10.1245/s10434-013-3070-y. 23800896

[30] Kojima K, April C, Canasto-Chibuque C, Chen X, Deshmukh M, Venkatesh A, et al. Transcriptome profiling of archived sectioned formalin-fixed paraffin-embedded (AS-FFPE) tissue for disease classification. PloS One. 2014;9(1):e86961. 10.1371/journal.pone.0086961. PMC3907407 24498002

[31] Villa E, Critelli R, Lei B, Marzocchi G, Camma C, Giannelli G, et al. Neoangiogenesis-related genes are hallmarks of fast-growing hepatocellular carcinomas and worst survival. Results from a prospective study. Gut. 2016;65(5):861–9. 10.1136/gutjnl-2014-308483. 25666192

[32] Villanueva A, Portela A, Sayols S, Battiston C, Hoshida Y, Mendez-Gonzalez J, et al. DNA methylation-based prognosis and epidrivers in hepatocellular carcinoma. Hepatology. 2015;61(6):1945–56. 10.1002/hep.27732. PMC12337117 25645722

[33] Grinchuk OV, Yenamandra SP, Iyer R, Singh M, Lee HK, Lim KH, et al. Tumor-adjacent tissue co-expression profile analysis reveals pro-oncogenic ribosomal gene signature for prognosis of resectable hepatocellular carcinoma. Mol Oncol. 2018;12(1):89–113. 10.1002/1878-0261.12153. PMC5748488 29117471

[34] Zhang J, Baran J, Cros A, Guberman JM, Haider S, Hsu J, et al. International Cancer Genome Consortium Data Portal--a one-stop shop for cancer genomics data. Database (Oxford). 2011;2011:bar026. 10.1093/database/bar026. PMC3263593 21930502

[35] Barrett T, Wilhite SE, Ledoux P, Evangelista C, Kim IF, Tomashevsky M, et al. NCBI GEO: archive for functional genomics data sets – update. Nucleic Acids Res. 2013;41(D1):D991–5. 10.1093/nar/gks1193. PMC3531084 23193258

[36] Lian Q, Wang S, Zhang G, Wang D, Luo G, Tang J, et al. HCCDB: a database of hepatocellular carcinoma expression atlas. Genom Proteom Bioinform. 2018;16(4):269–75. 10.1016/j.gpb.2018.07.003. PMC6205074 30266410

[37] Roessler S, Lin G, Forgues M, Budhu A, Hoover S, Simpson RM, et al. Integrative genomic and transcriptomic characterization of matched primary and metastatic liver and colorectal carcinoma. Int J Bio Sci. 2015;11(1):88–98. 10.7150/ijbs.10583. PMC4278258 25552933

[38] Ye QH, Qin LX, Forgues M, He P, Kim JW, Peng AC, et al. Predicting hepatitis B virus-positive metastatic hepatocellular carcinomas using gene expression profiling and supervised machine learning. Nat Med. 2003;9(4):416–23. 10.1038/nm843. 12640447

[39] Liao R, Wu H, Yi Y, Wang JX, Cai XY, He HW, et al. Clinical significance and gene expression study of human hepatic stellate cells in HBV related-hepatocellular carcinoma. J Exp Clin Cancer Res. 2013;32:22. 10.1186/1756-9966-32-22. PMC3654985 23601182

[40] Zhao S, Zhou L, Niu G, Li Y, Zhao D, Zeng H. Differential regulation of orphan nuclear receptor TR3 transcript variants by novel vascular growth factor signaling pathways. FASEB J. 2014;28(10):4524–33. 10.1096/fj.13-248401. PMC4202099 25016027

[41] Niu G, Ye T, Qin L, Bourbon PM, Chang C, Zhao S, et al. Orphan nuclear receptor TR3/Nur77 improves wound healing by upregulating the expression of integrin beta4. FASEB J. 2015;29(1):131–40. 10.1096/fj.14-257550. PMC4285545 25326539

[42] Tsuchida T, Friedman SL. Mechanisms of hepatic stellate cell activation. Nat Rev Gastroenterol Hepatol. 2017;14(7):397–411. 10.1038/nrgastro.2017.38. 28487545

[43] Peng L, Yuan XQ, Zhang CY, Ye F, Zhou HF, Li WL, et al. High TGF-beta1 expression predicts poor disease prognosis in hepatocellular carcinoma patients. Oncotarget. 2017;8(21):34387–97. 10.18632/oncotarget.16166. PMC5470976 28415739

[44] Zavadil J, Bottinger EP. TGF-beta and epithelial-to-mesenchymal transitions. Oncogene. 2005;24(37):5764–74. 10.1038/sj.onc.1208927. 16123809

[45] Kalluri R, Weinberg RA. The basics of epithelial–mesenchymal transition. J Clin Invest. 2009;119(6):1420–8. 10.1172/JCI39104. PMC2689101 19487818

[46] Lamiche C, Clarhaut J, Strale PO, Crespin S, Pedretti N, Bernard FX, et al. The gap junction protein Cx43 is involved in the bone-targeted metastatic behaviour of human prostate cancer cells. Clin Exp Metastasis. 2012;29(2):111–22. 10.1007/s10585-011-9434-4. 22080401

[47] Ogawa K, Pitchakarn P, Suzuki S, Chewonarin T, Tang M, Takahashi S, et al. Silencing of connexin 43 suppresses invasion, migration and lung metastasis of rat hepatocellular carcinoma cells. Cancer Sci. 2012;103(5):860–7. 10.1111/j.1349-7006.2012.02228.x. PMC7659240 22320152

[48] Imai Y, Yoshida O, Watanabe T, Yukimoto A, Koizumi Y, Ikeda Y, et al. Stimulated hepatic stellate cell promotes progression of hepatocellular carcinoma due to protein kinase R activation. PloS one. 2019;14(2):e0212589. 10.1371/journal.pone.0212589. PMC6386440 30794626

[49] Lv X, Fang C, Yin R, Qiao B, Shang R, Wang J, et al. Agrin para-secreted by PDGF-activated human hepatic stellate cells promotes hepatocarcinogenesis in vitro and in vivo. Oncotarget. 2017;8(62):105340–55. 10.18632/oncotarget.22186. PMC5739642 29285255

[50] Xie YX, Liao R, Pan L, Du CY. ERK pathway activation contributes to the tumor-promoting effects of hepatic stellate cells in hepatocellular carcinoma. Immunol Lett. 2017;188:116–23. 10.1016/j.imlet.2017.06.009. 28668554

[51] Rawal P, Siddiqui H, Hassan M, Choudhary MC, Tripathi DM, Nain V, et al. Endothelial cell-derived TGF-beta promotes epithelial–mesenchymal transition via CD133 in HBx-infected hepatoma cells. Front Oncol. 2019;9:308. 10.3389/fonc.2019.00308. PMC6491671 31069171

[52] Liu WT, Jing YY, Yu GF, Chen H, Han ZP, Yu DD, et al. Hepatic stellate cell promoted hepatoma cell invasion via the HGF/c-Met signaling pathway regulated by p53. Cell Cycle. 2016;15(7):886–94. 10.1080/15384101.2016.1152428. PMC4889302 27077227

[53] Chen YC, Chang HM, Cheng JC, Tsai HD, Wu CH, Leung PC. Transforming growth factor-beta1 up-regulates connexin43 expression in human granulosa cells. Hum Reprod. 2015;30(9):2190–1. 10.1093/humrep/dev175. PMC4542719 26202915

[54] Qiu X, Cheng JC, Zhao J, Chang HM, Leung PC. Transforming growth factor-beta stimulates human ovarian cancer cell migration by up-regulating connexin43 expression via Smad2/3 signaling. Cell Signal. 2015;27(10):1956–62. 10.1016/j.cellsig.2015.07.010. 26186970

[55] Lim MC, Maubach G, Zhuo L. TGF-beta1 down-regulates connexin 43 expression and gap junction intercellular communication in rat hepatic stellate cells. Eur J Cell Biol. 2009;88(12):719–30. 10.1016/j.ejcb.2009.08.003. 19781809

[56] James CC, Zeitz MJ, Calhoun PJ, Lamouille S, Smyth JW. Altered translation initiation of GJA1 limits gap junction formation during epithelial–mesenchymal transition. Mol Biol Cell. 2018;29(7):797–808. 10.1091/mbc.E17-06-0406. PMC5905293 29467255

